# Promoting mental health in intensive care unit onboarding: a consensus-based iterative development of a competency-based framework

**DOI:** 10.3389/fmed.2026.1872115

**Published:** 2026-07-21

**Authors:** Tobias Bexten, Janina Henneberg, David Josuttis, Anne Kamphausen, Dominik Hinzmann

**Affiliations:** 1 Department for Interdisciplinary Intensive Medicine and Intermediate Care, Helios Dr. Horst Schmidt Kliniken Wiesbaden, Wiesbaden, Germany; 2 German Interdisciplinary Association for Intensive Care and Emergency Medicine (DIVI), Berlin, Germany; 3 Department of Pulmonology and Respiratory Medicine, Klinikum Ernst von Bergmann Potsdam, Potsdam, Germany; 4 Department of Nephrology and Medical Intensive Care, Charité – Universitätsmedizin Berlin, Berlin, Germany; 5 Department of Anesthesiology and Surgical Intensive Care Medicine, Nuremberg General Hospital, Paracelsus Medical University, Nuremberg, Germany; 6 Department of Anesthesiology and Intensive Care Medicine, TUM School of Medicine and Health, Munich, Germany

**Keywords:** competency-based medical education, health professions education, intensive care unit onboarding, mental health promotion, second victim phenomenon

## Abstract

**Background:**

Healthcare professionals in intensive care units face substantial psychological stress, yet structured educational approaches to promote mental health during early training remain underdeveloped. While current recommendations advocate early integration of psychosocial support into onboarding, they have not been translated into competency-based educational frameworks aligned with modern health professions education.

**Objective:**

To develop a competency-based educational framework for ICU onboarding that integrates mental health promotion into structured learning objectives across knowledge, reflection, and action domains.

**Methods:**

A mixed-methods design was applied, combining a structured consensus process with competency-based curricular mapping. Guiding questions were iteratively refined using a two-stage rapid consensus process involving a multidisciplinary panel of clinicians, educators, and external experts in psychology and occupational health. Consensus was defined as the absence of substantial disagreement. The resulting content was mapped to competency dimensions informed by competency-based medical education principles and aligned with Miller’s Pyramid, a hierarchical model of clinical competence ranging from knowledge to action.

**Results:**

The process resulted in a competency-based framework comprising eight educational domains: relevance of mental health, ICU-specific stressors, conceptual understanding of stress, resilience and coping, the second victim phenomenon (the psychological impact on healthcare professionals following adverse events), structural support systems, professional self-reflection, and shared responsibility. Each domain was operationalized into competency levels (knowledge to evaluation), enabling constructive alignment between learning objectives, teaching formats, and potential assessment strategies. The framework integrates individual, team, and organizational perspectives and provides a structured basis for embedding psychosocial competencies into onboarding curricula.

**Conclusion:**

This study may contribute to advancing health professions education and supports constructive alignment among learning objectives, teaching formats, and assessment strategies. It operationalizes existing recommendations into actionable educational components and supports constructive alignment in curriculum design. Implementation and evaluation studies are warranted to assess its impact on learning outcomes, behavioral change, and clinical practice.

## Background

Healthcare professionals working in intensive care units (ICUs) are exposed to a substantially increased risk of work-related psychological stress (1).

High workload, exposure to potentially traumatic events, and chronic strain are associated with elevated rates of burnout and post-traumatic stress reactions (2–4). Reported burnout prevalence reaches up to 59.4%, accompanied by a high burden of clinically relevant stress symptoms (5, 6).

These findings underline the need for early, preventive, and structurally embedded in-house support systems (7). Corresponding recommendations have been outlined in the “Recommendations on the Structure and Equipment of Intensive Care Units” by the DIVI (German Interdisciplinary Association for Intensive Care and Emergency Medicine) sections “Perspective Resilience” and “Psychological Care Structures in Intensive Care Medicine.” These emphasize the implementation of psychosocial support structures for healthcare staff, including trained peer support as a central component (D.6.2 Personnel care: psychosocial support and strengthening of resilience) (8).

Core messages of these recommendations highlight that psychosocial support for ICU staff represents a key determinant of both staff wellbeing and patient safety (7, 9).

In particular, low-threshold, process-oriented interventions, such as peer support programs, have demonstrated effectiveness in reducing psychological burden, even as stand-alone measures (3). In addition, experiences from pandemic and crisis settings underscore the importance of easily accessible support services and the provision of basic needs as essential elements of sustainable psychosocial care (10).

This need is further reflected in the Young DIVI position paper on onboarding in intensive care medicine (the junior members’ network of the German Interdisciplinary Association for Intensive Care and Emergency Medicine). Recommendation 9 explicitly states that measures to promote mental health should be established during the orientation period of new professionals (9). Early integration of supportive concepts, clearly defined contact persons, and low-threshold access to support services is considered crucial to sustainably promote mental health and to reduce barriers to help-seeking in situations of negative stress (8). To date, systematic data on the prevalence and format of mental health integration in ICU onboarding programs are lacking. Where such integration exists, it typically consists of brief, didactic formats, such as single informational sessions or online modules, rather than structured, competency-based curricula embedded in a longitudinal onboarding process (10).

Despite this, a framework for translating psychosocial support principles into actionable learning objectives for ICU onboarding is absent from the literature. Existing approaches either focus on individual interventions or address organizational structures in isolation, without providing a unified curricular basis for early career professionals.

Internationally, efforts to integrate wellbeing into medical education were already proposed: the ACGME Common Program Requirements mandate that residency programs in the United States address burnout, depression, and mental health access, and a longitudinal wellness curriculum was developed following the 2017 Resident Wellness Consensus Summit (11, 12).

However, these approaches are not ICU-specific, are not structured around competency-based curricular mapping, and do not address the distinct psychosocial demands of intensive care onboarding.

Our study addresses this gap by developing and operationalizing a competency-based framework that integrates mental health promotion into ICU onboarding. Thus, it may contribute to advancing health professions education and supports constructive alignment among learning objectives, teaching formats, and assessment strategies.

### Objective

The objective of this study is to develop and operationalize a competency-based framework comprising knowledge, reflection, and action competencies for onboarding in intensive care medicine. The framework aims to facilitate structured knowledge transfer and to provide a curricular basis for mental health promotion among healthcare professionals during early career stages in the ICU.

### Research question

Which knowledge, reflection, and action competencies should healthcare professionals acquire during onboarding in intensive care units to maintain and promote mental health in the context of their professional practice?

## Methodology

The consensus process comprised two sequential rounds. In Round 1, two authors (T.B. and J.H.) developed an initial set of thematic domains and guiding questions through iterative synthesis of clinical experience and a targeted literature review. This draft framework was subsequently circulated asynchronously to the full author panel for structured review and written comment. In both rounds, the consensus process was directed by the study’s guiding question: “Which knowledge, reflection, and action competencies should healthcare professionals acquire during onboarding in intensive care units to maintain and promote mental health in the context of their professional practice?” focusing on three criteria: (1) clarity of wording, (2) relevance to mental health promotion during ICU onboarding, and (3) applicability within existing onboarding structures. Responses were provided as open-ended written comments. Disagreements were resolved through moderation and direct bilateral exchange between the coordinating authors and dissenting panel members, with iterations continuing until agreement was reached. In Round 2, the revised framework was circulated asynchronously to the six external experts using the same procedure. Consensus was defined as the absence of substantive objections following each round. Specifically, an item was considered consensual if no panel member objected to its inclusion after the opportunity for bilateral discussion and revision. No formal voting or quantitative threshold was applied, consistent with the exploratory and framework-development nature of this study.

The author panel consisted of five clinician-educators with expertise in intensive care medicine, anesthesiology, emergency medicine, intensive care nursing, and medical education, all of whom were actively involved in onboarding and curricular development. Six external experts were additionally recruited through deliberate selection. Inclusion criteria were: (1) professional experience in teaching or educational contexts; (2) professional experience in onboarding processes; and (3) professional experience in psychosocial support.

The process was conducted in two iterative rounds between January and April 2026. Competency mapping followed the competency dimensions (e.g., knowledge, assessment, evaluation, adaptation), guided by the Young DIVI’s position paper on structured training in intensive care medicine and Miller’s Pyramid, a hierarchical model of clinical competence ranging from knowledge to action (13). Here, initial domain-competency assignments were proposed by the coordinating authors (T.B. and J.H.) as part of the draft framework and were refined iteratively across both consensus rounds by the full panel. Mapping decisions were thus embedded within the consensus process itself rather than conducted as a separate coding procedure.

The Glossary is provided in the Supplementary material.

### Ethics statement

The study protocol was submitted to the Ethics Committee of the State Medical Association of Hesse (Landesärztekammer Hessen), which issued a formal waiver stating that no ethics approval was required for this project (Reference No. 2026-4462-AF). External experts participated solely in their professional capacity to review and refine the framework, and no personal data were collected or analyzed.

## Results

*Panel composition*: Eleven panel members participated in the consensus process: five clinician-educators forming the author panel and six external experts. The resulting external expert panel covered a broad range of fields, including psychology and trauma therapy, occupational health, pediatric intensive care nursing, and community healthcare. These external experts were recruited from diverse institutional settings, including university hospitals, universities of applied sciences, and maximum care centers, reflecting a broad range of professional contexts and practice environments across Germany. All panel members participated in both consensus rounds as applicable; no panel member withdrew during the process.

The results are divided into Sections 3.1 and 3.2. Section 3.1 provides an overview of the key questions and areas of expertise, while Section 3.2 outlines the basic concept in more detail.

### Key questions and areas of expertise

The consensus-based development process resulted in a structured competency framework comprising eight core domains related to mental health in intensive care unit onboarding. These domains cover the relevance of mental health, ICU-specific stressors, conceptual understanding of stress, resilience and coping strategies, the second victim phenomenon, structural support services, professional self-reflection, and shared responsibility.

**Figure 1:**
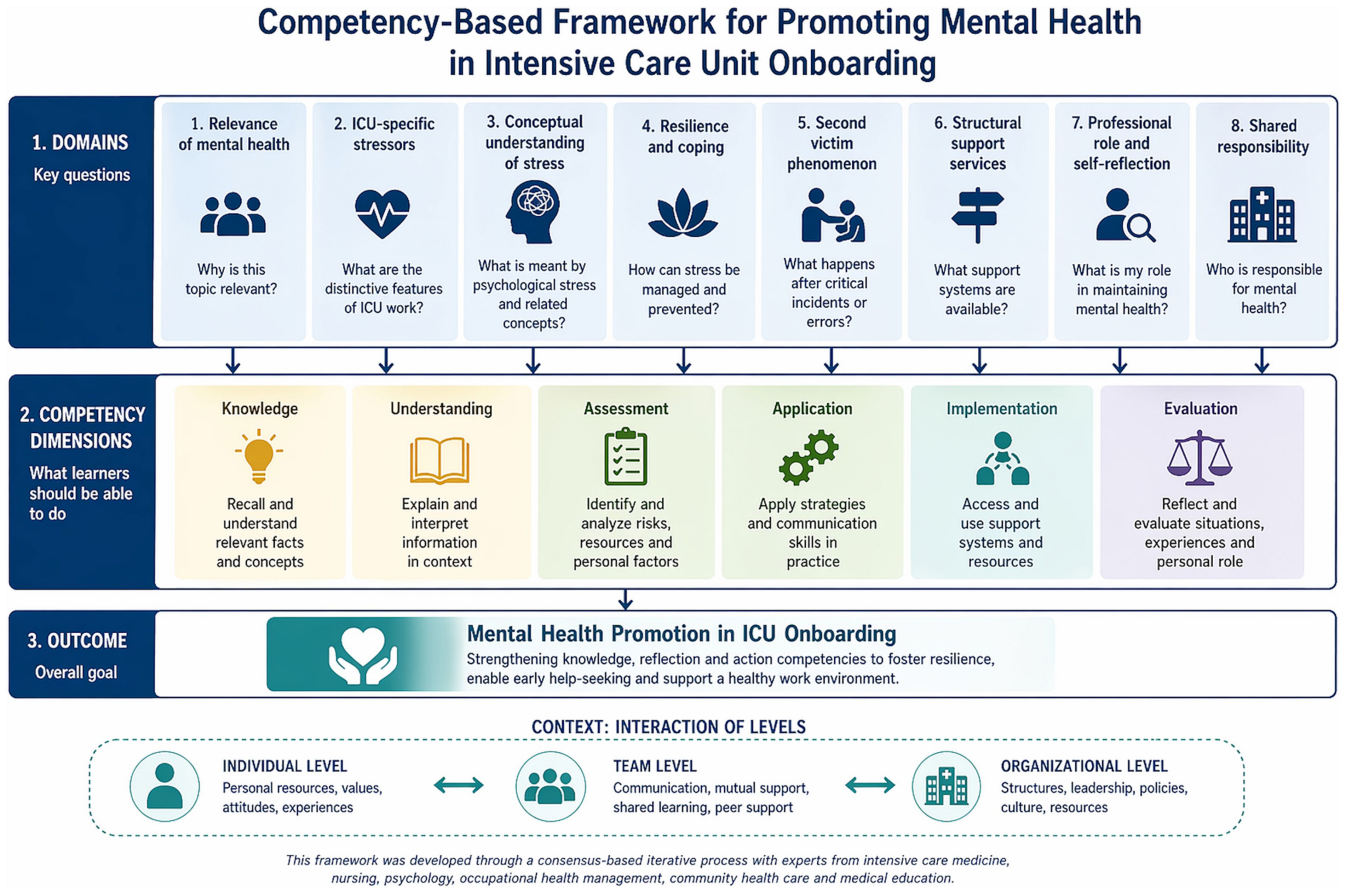
Competency-based framework for promoting mental health in intensive care unit onboarding. The model integrates eight core domains with competency dimensions (knowledge to evaluation) (13, 41) and highlights the interaction between individual, team, and organizational levels.

Each domain was mapped to specific competency dimensions, including knowledge, understanding, assessment, application, implementation, and evaluation. A detailed overview of key questions and exemplary competencies is provided in Table 1 and Figure 1, including competency levels according to Miller’s pyramid. A full-text version of the competency framework, including the operational competency levels that were used by the Joung-DIVI is provided in the Supplementary material.

**Table 1:** Competency dimensions promoting mental health in ICU onboarding.

Domain	Key question	Competency dimension	Example competencies
1. Relevance of mental health	Why is this topic relevant?	Knowledge/understanding	Understand the relationship between employee mental health, patient safety, and staff retention
2. ICU-specific stressors	What are the distinctive features of ICU work?	Knowledge/understanding	Recognize setting-specific risks, including high workload, time pressure, and exposure to potentially traumatic events
3. Conceptual understanding of stress	What is meant by psychological stress and related concepts?	Knowledge/understanding	Differentiate between acute stress reactions, burnout, and post-traumatic stress disorder; understand key occupational psychology concepts
4. Resilience and coping	How can resilience act as a preventive factor?	Understanding/assessment/application	Identify individual risk and protective factors; apply strategies to strengthen personal and team resilience
5. Second victim phenomenon	What happens after critical incidents or errors?	Knowledge/application	Understand the concept of the second victim and recognize the need for support following adverse events
6. Structural support services	What support systems are available?	Knowledge/implementation	Identify and access institutional support services; understand reporting pathways and organizational measures
7. Professional role and self-reflection	What is my role in maintaining mental health?	Assessment/evaluation	Reflect on personal stress experiences and professional role; evaluate stressful and supportive situations
8. Shared responsibility	Who is responsible for mental health?	Knowledge	Understand the shared responsibility between individuals and organizations for maintaining a healthy work environment

Competency dimensions for promoting mental health in ICU onboarding. Domains and key questions were derived through a two-stage rapid consensus process involving a multidisciplinary author panel and six external experts. Competency levels are based on Miller’s Pyramid (knowledge, understanding, application, evaluation). ICU, intensive care unit; SVP, second victim phenomenon.

### Basic concept

Section 3.2 provides a summarized overview of the content for each key question.

1. Why is this topic relevant?

Employee mental health is a key determinant of patient safety, performance, and staff retention in intensive care, as high stress and burnout are associated with increased errors and turnover. Furthermore, presenteeism and absenteeism should also be considered, as they are becoming increasingly relevant, particularly in acute care settings. Early integration of psychosocial competencies during onboarding helps prevent stress-related outcomes and supports long-term professional stability (14, 15).

2. What are the distinctive features of ICU work?

ICUs are characterized by high complexity, time pressure, ethical dilemmas, and frequent exposure to critical and end-of-life situations, all of which contribute to substantial psychological stress. Additionally, technical demands, decision-making uncertainty, and error-prone environments further increase the burden on staff (8, 16).

3. What is meant by psychological stress and related concepts?

Psychological stress refers to external demands affecting individuals, while strain describes the individual response, which can vary widely. Acute stress reactions are short-term and self-limiting, whereas burnout and PTSD represent longer-term, clinically relevant consequences requiring different management approaches. While burnout reflects a chronic depletion of emotional and professional resources, moral distress arises when clinicians are prevented from acting in accordance with their ethical values, whereas compassion fatigue results from the cumulative burden of empathic engagement with suffering patients. Although distinct in their etiology, these constructs share overlapping consequences for staff wellbeing and patient safety, and an understanding of their differences supports targeted prevention and intervention. Understanding these concepts supports both individual coping and the development of supportive, learning-oriented work environments (5, 17–21).

4. How can resilience act as a preventive factor?

Resilience is a dynamic process involving individual and organizational factors that enables healthcare professionals to cope with stress and maintain wellbeing. Early resilience training during onboarding has both preventive and health-promoting effects by strengthening coping strategies and reducing vulnerability to stress (2, 22–24).

5. What happens after critical incidents or errors?

The second victim phenomenon describes the psychological impact on healthcare professionals following adverse events, which can lead to emotional distress, reduced performance, and increased turnover. Early recognition and structured support are essential to mitigate these effects and maintain both staff wellbeing and patient safety (25, 26).

6. What support systems are available?

Structured support systems, such as peer support, debriefings, supervision, and reporting pathways, provide essential mechanisms for coping with stressful events and preventing long-term consequences. Effective use of these services requires awareness, accessibility, and a supportive organizational culture that reduces barriers to help-seeking (27–30).

7. What is my role in maintaining mental health?

Self-reflection enables healthcare professionals to process experiences, recognize stress early, and develop a stable professional identity in a highly demanding environment. At the individual level, this may include maintaining personal boundaries, engaging in regular self-reflection, and proactively seeking support when needed. Structured reflection and mentoring during onboarding support resilience, learning, and interprofessional collaboration (31).

8. Who is responsible for mental health?

Mental health in intensive care results from the interaction between individual responsibility and organizational structures, both of which are essential for sustainable wellbeing and safe patient care. While individuals must actively engage in self-care and reflection, organizations must provide supportive structures, resources, and a culture of psychological safety (32). Here, organizational responsibilities may include peer support programs, structured debriefing formats, transparent reporting pathways, access to occupational health services, and a leadership culture that actively normalizes help-seeking behavior.

## Discussion

This study develops a structured, competency-based framework for onboarding in intensive care units with the aim of maintaining and promoting the mental health of new professionals. This addresses an existing gap between general recommendations for psychosocial support and their concrete curricular implementation. While previous approaches have often focused on individual interventions or structural measures (10, 33, 34), the model presented here systematically integrates knowledge, reflection, and action competencies into a unified framework.

The framework’s structure suggests that the promotion of mental health in intensive care medicine cannot be understood exclusively as an individual issue, but is situated at the intersection of individual resilience, team dynamics, and organizational conditions (35). In particular, the linking of concepts from occupational psychology (e.g., moral distress, compassion fatigue, second victim) with concrete action competencies is relevant to practice in this context (7, 36–39).

Although the framework was developed within the German healthcare context, the underlying challenges are international. In the United States, the ACGME Common Program Requirements oblige residency programs to address wellbeing, burnout, and access to mental healthcare, and a longitudinal resident wellness curriculum was developed following the 2017 Resident Wellness Consensus Summit (11, 12). At the European level, the ERNST consortium has advanced research and support structures for second victims across multiple countries (26, 28), and experiences from Sweden demonstrate the rapid implementation of psychological support models for frontline staff during the COVID-19 pandemic (10).

None of these approaches, however, offers an ICU-specific, competency-based onboarding curriculum. The framework presented here may therefore also inform international efforts, pending cross-cultural adaptation and validation in other healthcare systems.

It addresses all professional categories newly entering the ICU, whether in early specialty training or already specialized, within the onboarding period.

The consensus-based multi-professional development approach ensured that the content was highly relevant, incorporating diverse professional perspectives from intensive care medicine, nursing, community health, occupational and community healthcare, and psychosocial care. At the same time, competency mapping is intended to facilitate integration into existing orientation concepts and curricula, pending empirical evaluation (40).

### Limitations

This study has several limitations. First, the consensus process was conducted within a relatively small group of experts, which may limit the generalizability of the findings despite the inclusion of multidisciplinary perspectives. In addition, demographic characteristics of the panel members (e.g., age, gender, time since graduation) were not systematically collected, which limits the transparency of panel reporting. Panel expertise is therefore described qualitatively by professional category, field of expertise, and institutional setting.

Second, although external experts were involved, international validation of the framework is still pending.

Third, defining consensus as the absence of substantive objections carries a risk of selection bias. Moreover, the non-anonymous, moderated format cannot exclude social desirability. The absence of formal voting may have also influenced which domains and competencies were ultimately included. The framework should therefore be regarded as a preliminary consensus product, whose content validity requires confirmation through more formal methods (e.g., a Delphi study with quantitative agreement criteria) during the planned validation stage.

Fourth, the framework represents a conceptual model and has not yet been empirically tested in clinical practice.

Finally, the transferability of the framework to different healthcare systems and professional contexts may be limited and requires further evaluation.

## Conclusion and outlook

The competency-based framework developed here provides a structured and practical foundation for integrating psychosocial aspects into orientation and onboarding programs in intensive care units. It enables the systematic development of teaching content that can be adapted to local institutional contexts and professional cultures, and may support healthcare professionals in identifying stressors early, facilitating access to support services, and potentially strengthening both employee wellbeing and patient safety, in the long term.

Future studies should further elaborate this framework in terms of content, translate it into practical formats (e.g., handouts), and, in particular, monitor and evaluate its implementation in clinical practice. To our understanding, validation studies should employ a stepwise pilot approach that focuses on outcome metrics such as burnout scores and help-seeking behavior to evaluate the framework’s feasibility and impact on learning outcomes.

## Data Availability

The original contributions presented in the study are included in the article/Supplementary material, further inquiries can be directed to the corresponding author.
