# Supplementary material for: Promoting mental health in intensive care unit onboarding: a consensus-based iterative development of a competency-based framework
Source: Front Med (Lausanne). 2026 Jul 21;13:1872115. doi: 10.3389/fmed.2026.1872115 (PMC13434846; doi:10.3389/fmed.2026.1872115)
Supplement: Supplementary file 1 [file Supplementary_file_1.pdf]

Promoting Mental Health in Intensive Care Unit Onboarding:
A Consensus-Based Iterative Development of a Competency-Based Framework

Supplement:

S1. Glossary

Basic terms for describing the competencies to be achieved, based on the structure of the appendix “Induction into Intensive Care Medicine” (9).

Knowledge: Encompasses theoretical expertise in a subject area as well as the ability to identify and classify fundamental content.

Orientation / Basic Understanding: Describes an initial understanding of a subject area in the sense of a first classification of content without the expectation of in-depth evaluation or application.

Information Transfer: Encompasses the structured communication of relevant content within the framework of orientation. The goal is to establish a basic understanding of topics, terms, and relationships.

Preparation: Refers to the structured preparation of materials, procedures, or patients in the context of a planned intervention.

Assistance: Describes supportive participation in procedures that may not be performed independently.

Application: Involves the safe use of materials, equipment, or procedures. This requires knowledge of potential risks, complications, and measures for their prevention and early detection.

Implementation: Refers to the independent practical implementation of a procedure in accordance with professional standards.

Assessment: Encompasses the structured evaluation of clinical, diagnostic, and contextual information with the aim of identifying risks early and deriving appropriate measures.

Evaluation: Describes the systematic assessment of outcomes, effects, and potential complications of a performed measure.

Adjustment: Involves deriving and implementing follow-up measures based on the evaluation.

Indication / Recognition: Refers to the independent assessment of the necessity of a measure based on the clinical situation.

Implementation under professional supervision: Implementation, assessment, evaluation, and adjustment are carried out under the guidance and responsibility of a qualified professional (e.g., a nurse with advanced training in intensive care and anesthesia nursing or a medical professional). Implementation takes place within the framework of interprofessional coordination.
