# Supplementary material for: Promoting mental health in intensive care unit onboarding: a consensus-based iterative development of a competency-based framework
Source: Front Med (Lausanne). 2026 Jul 21;13:1872115. doi: 10.3389/fmed.2026.1872115 (PMC13434846; doi:10.3389/fmed.2026.1872115)
Supplement: Supplementary file 2 [file Supplementary_file_2.pdf]

Promoting Mental Health in Intensive Care Unit Onboarding:
A Consensus-Based Iterative Development of a Competency-Based Framework

***Supplement:* S2. Detailed competency framework**

1. **Relevance of mental health:**
    1.1. Why is this topic relevant? Employee health as a factor in patient safety; staff shortages/turnover/leaving the profession due to mental overload
        1.1.1. **Understands** the connections between employee health, performance, and quality of care, as well as job satisfaction
        1.1.2. **Receives information** on the incidence of secondary illnesses resulting from work-related stress.

2. **ICU-specific stressors:**
    2.1. What are the distinctive features of intensive care units
        2.1.1. **Understands** potentially traumatic events and setting-specific risks
        2.1.2. **Receives information** on the types of psychological stress that may arise

3. **Conceptual understanding of stress:**
    3.1. What is meant by psychological strain / psychological stress, acute stress reaction, compared to burnout and PTSD

    3.2. What is meant by Occupational psychology terms (Culture of Safety, Organizational Betrayal, Just Culture, Moral Distress, Moral Injury, Compassion Fatigue, Decision Fatigue)

        3.2.1. **Receives information** on the terms

        3.2.2. **Receives information** in the form of illustrative case studies

4. **Resilience and coping:**
    4.1. How can Resilience be a preventive factor
        4.1.1. **Understands** the concept of resilience and the model of risk and protective factors
        4.1.2. **Is familiar with** specific protective factors of resilience as they relate to their work.
        4.1.3. **Assesses** risks and protective factors in relation to their own work and understands how a balance between them can be achieved or restored.
        4.1.4. **Receives information** on ways to strengthen and maintain their own resilience as well as resilience within the team.

- 4.1.5. **Understands** the interplay or interdependence between individual, team, and organizational resilience.
- 4.1.6. **Is familiar with** programs and measures to strengthen resilience within the organization (e.g., health promotion, psychosocial support services, personal agency, and possible measures)

5. The Second Victim Phenomenon:

5.1. What happens after critical incidents or errors?

- 5.1.1. **Understands** the term "second victim"
- 5.1.2. **Receives information** on the Second Victim phenomenon as well as potential support services

6. Structural support services:

6.1. What support systems are available?

- 6.1.1. **Identification/recognition** of a possible course of action following particularly stressful events and **knowledge** of how and where these are correctly documented (keyword: incident report form, accident report).
- 6.1.2. **Receives information** on the levels of support according to ERNST (European Researchers' Network Working On Second Victims) and the differences between them
- 6.1.3. **Guidance** on reporting as a 'PSYCH work-related accident' and care in accordance with DGUV
- 6.1.4. **Receives information** on practical interventions in an organizational context
- 6.1.5. **Receives information** in the form of illustrative case examples
- 6.1.6. **Implementation**: Actively seek support and identify potential obstacles

7. **Professional role and self-reflection:** What is my role in maintaining mental health

7.1. Self-reflection

- 7.1.1. **Assessment** (self-reflection and reflection on one's role)
- 7.1.2. **Evaluation** (What was stressful/helpful)

8. **Shared responsibility:** Who is responsible for mental health?

8.1. What responsibility do I bear myself / What does the organization bear

- 8.1.1. **Receives information** about their own responsibilities and those of the organization
- 8.1.2. **Receives information** about opportunities for participation
